# Prevalence of Intestinal Parasitic Infections and Their Associated Risk Factors among Pregnant Women Attending Antenatal Care Center at Woreilu Health Center, Woreilu, Northeast Ethiopia

**DOI:** 10.1155/2022/5242252

**Published:** 2022-04-11

**Authors:** Edosa Kebede, Netsanet Asefa, Chala Daba, Daniel Gebretsadik

**Affiliations:** ^1^Department of Medical Laboratory Science, College of Medicine and Health Sciences, Ambo University, Ambo, Ethiopia; ^2^Department of Medical Laboratory Science, College of Medicine and Health Sciences, Wollo University, Dessie, Ethiopia; ^3^Department of Medical Laboratory Science, Woreilu Health Center, Woreilu, Ethiopia; ^4^Department of Environmental Health Science, College of Medicine and Health Sciences, Wollo University, Dessie, Ethiopia

## Abstract

**Background:**

Intestinal parasitic infections (IPIs) affect millions of pregnant women worldwide and if left untreated can cause adverse effects for mothers, fetuses, and newborns. This study was aimed at determining the prevalence and associated risk factors of intestinal parasitic infections among pregnant women in Woreilu, Northeast Ethiopia.

**Methods:**

A cross-sectional study was conducted in Woreilu Health Center from October 2018 to February 2019. A convenient sampling technique was used to select the study subjects. The data related to the sociodemographic information and associated risk factors were collected by the interview technique, and fresh stool samples were collected from each pregnant woman. The microscopic examination of the stool samples was done by using direct wet mount preparations in normal saline and formol ether concentration techniques. Descriptive statistics and binary logistic regression were used. A *p* value < 0.05 was taken as statistically significant.

**Results:**

A total of 331 pregnant women were included. The mean ± standard deviation of age of the participants was 26.3 ± 5.96 years ranging between 16 and 43 years. The overall prevalence of IPIs was 144 (43.5%) with the predominance of *E. histolytica/dispar* (44.4%) followed by *A. lumbricoides* (15.7%). Being a student (AOR = 3.35, 95% CI: 1.01-11.09, *p* = 0.047), second trimester (AOR = 3.94, 95% CI: 1.46-10.64, *p* = 0.007), third trimester (AOR = 3.32, 95% CI: 1.15-9.6, *p* = 0.027), and using spring water for drinking (AOR = 2.91, 95% CI: 1.62-5.22, *p* ≤ 0.001) were significantly associated with IPIs.

**Conclusion:**

The prevalence of IPIs was high in this study. Being a student, second and third trimester, and using spring water for drinking were associated factors with IPIs. Therefore, improving the sanitation of the students and providing clean drinking water for the women should be strengthened. Screening women and providing health education during their antenatal care (ANC) visits are also recommended.

## 1. Introduction

Intestinal parasitic infections (IPIs) caused by protozoa and helminthes are common problems in developing countries and are of major health problem due to their high prevalence rate and effect on both nutritional and immune status of the population [1, 2].

According to a recent report by the World Health Organization (WHO), about 1.5 billion people (24% of the global population) are infected with intestinal parasites [3]. IPIs are widely distributed in tropical and subtropical areas, with the greatest numbers occurring in sub-Saharan Africa, the Americas, China, and East Asia [3]. The major IPI of global public health concern is the protozoan species like *Entamoeba histolytica* and *Giardia lamblia* and helminthic species like *Ascaris lumbricoides*, *Trichuris trichiura*, hookworm species, *Enterobius vermicularis*, *Taenia* species, and *Schistosoma mansoni* [2, 4].

Parasitic infections are common among pregnant women due to the reduced body immunity and therefore can affect physiological systems of the body [5]. Moreover, it is also indicated that pregnant women with IPIs are at increased risk of maternal complications and adverse perinatal outcomes such as anemia, poor fetal growth, low birth weight, and perinatal mortality [6–8]. IPIs may cause anemia by different mechanisms. Some may promote indigenous nutrient loss by inducing intestinal mucosa damage, impairing digestion, and causing diarrhea. *G. lamblia* and *E. histolytica* also cause blood loss and inflammation-induced restriction of iron absorption. Hookworms, schistosomiasis, and *T. trichiura* lead to intestinal blood loss, and others reduce appetite and compromise nutrient intake [9–11].

Those residents living under the poverty line in low-income countries are at a high risk of IPIs, especially pregnant women and their infants and children [12], and the WHO recommends that endemic countries practice periodic treatment (deworming) of at-risk people to reduce intestinal parasite-related morbidity [3]. In Ethiopia, IPIs are very common and the magnitude of infection varies from place to place which may be due to the unsafe and inadequate provision of water, unhygienic living conditions, the absence of proper utilization of latrine, and the habit of walking with barefoot [13–17].

The data regarding the overall prevalence of IPIs among pregnant women is scarce in Ethiopia. However, several studies demonstrated the very high prevalence of IPIs among pregnant women. For instance, the overall prevalence of IPIs among pregnant women attending ANC in Yifag Health Center, Northwest Ethiopia, was 53.4% [18]; that among pregnant women in West Gojjam Zone, Northwest Ethiopia, was 37.3% [19]; that among pregnant women attending prenatal care at Felege Hiwot Referral Hospital, Bahir Dar, was 31.5% [15]; that among pregnant women attending prenatal care in the Northwestern Ethiopia was 36.7% [20]; that among pregnant women in the Wondo Genet District, Southern Ethiopia, was 38.7% [16]; and that among pregnant women attending ANC at public health facilities in the Lalo Kile District, Oromia, Western Ethiopia, was 43.8% [21].

The habit of soil eating, being a farmer, lack of proper sanitation and hygiene, consumption of raw vegetables, walking barefoot, dwelling in a rural area, improper use of latrine, lack of health education, and water source were the factors associated with IPIs among pregnant women [18–22].

Even though there are many studies conducted in Ethiopia that have reported the magnitude of intestinal parasitic infections, the distribution and prevalence of intestinal parasites differ from region to region due to a range of different factors, and also, there remains a scarcity of published data among pregnant women, especially in the study area where adequate health facilities are limited.

Therefore, this study assessed the prevalence and associated risk factors of IPIs among pregnant women at Antenatal Care Center (ANC) of Woreilu Health Center, Northeast Ethiopia. The results of this study may provide evidence for stakeholders in health care and public health and are also important in implementing and improving preventive measures in pregnant women so the impact of IPIs during pregnancy could be minimized.

## 2. Methods and Material

### 2.1. Study Design, Period, and Area

A health institution-based cross-sectional study was conducted at ANC clinic of Woreilu Health Center from October 2018 to February 2019. Woreilu is one of the districts in South Wollo Zone of Amhara regional state which is found at 492 km far from Addis Ababa, Northeast Ethiopia, with latitude and longitude of 10°36′N and 39°26′E, respectively. The total population of Woreilu is 127,041. The district has a total of 24 kebeles (neighborhood units): 20 rural and 4 urban. In Woreilu District, there are 5 health centers and one district hospital [23]. This study was conducted in Woreilu Health Center which is one of the health centers in the district under the Woreilu health Office.

### 2.2. Study Population and Sample Size Determination

The source population was all pregnant women attending ANC clinic of Woreilu Health Center. The study population consisted of selected pregnant women attending ANC clinic at the health center during the study period and those who were residents in Woreilu Town and surrounding kebeles and have not received antiparasitic treatment for the last 1 month. The minimum sample size was calculated using a single population proportion formula with the assumption of 29.5% previous prevalence rate of intestinal parasitic infection among pregnant women [22], at a 95% level of confidence (*Z* = 1.96) and a margin of error (5%). In line with these conditions, the minimum number of study participants was 319, and by adding a 5% nonresponse rate, the final sample size was calculated to be 335. A convenient sampling technique was used to select the study subjects.

### 2.3. Inclusion and Exclusion Criteria

Pregnant women who were permanent residents in Woreilu and who were willing to participate in the study were included, whereas pregnant women who were taking antiparasitic drugs within one month of the data collection period and who were critically ill were excluded from the study.

### 2.4. Stool Specimen Collection and Examination

For specimen collection, a single well-labeled, clean, dry, disinfectant-free, wide-mouthed plastic container was distributed to each study participant with instruction requesting them to bring the stool sample immediately. The containers were labeled with the study participants' names, code numbers, and dates of collection. Stool samples were collected from each woman along with a questionnaire. After receiving about 5 g single stool specimen, the microscopic examination of the stool sample was done at the Woreilu Health Center laboratory by using the direct wet mount preparations in normal saline and formol ether concentration techniques [24].

### 2.5. Data Collection, Processing, and Analysis

An interview-based structured questionnaire was used to collect the sociodemographic and behavioral-related data from each study participant by the investigators. The items of the questionnaire were prepared by reviewing similar studies conducted elsewhere with minor modifications to suit the local context and objective of the present study. It was prepared in English and translated to the local language (Amharic) and then translated back into English to check the accuracy of the translation. Epi-Info version 3.5.1 was used to enter the data, and it was transferred to SPSS version 20 software for analysis. Summary results were presented by frequency tables. Furthermore, odds ratio, 95% confidence interval, and *p* value were computed using a binary logistic regression to investigate the association between the dependent and independent variables. A *p* value < 0.05 was considered statistically significant.

### 2.6. Data Quality Management

Experienced laboratory technologists with at least 2 years of work experience performed laboratory investigations. Slide cross-checking was done blindly by other laboratory technologists, and a standard operational procedure of examinations was followed. Careful cleaning, coding, and entering of data were done.

### 2.7. Ethical Considerations

The study was conducted after ethical clearance was obtained from the research ethics review committee of the College of Medicine and Health Sciences, Wollo University, and permission was also obtained from Woreilu Health Center. Verbal and written consent was obtained from all study participants before the data collection. The confidentiality of the study participants was also strictly maintained by using data without any personal identifier. Pregnant women with positive results were referred to the clinician in ANC clinic for antiparasitic treatment.

## 3. Results

### 3.1. Sociodemographic Characteristics of the Participants

A total of 331 pregnant women participated in this study. The age range of the study participants was from 16 to 43 with a mean age of 26.3 ± 5.96 years. About 112 (33.8%) of the study participants were within the age group of 20 to24 years. Nearly half (155, 46.8%) of pregnant women were rural dwellers. Regarding marital status, 273 (82.5%) were married, 39 (11.8%) were single, 14 (4.2%) were divorced, and the rest 5 (1.5%) were widowed. Out of the 331 pregnant women, 153 (46.3%) were housewives. The educational status of the study participants showed that 135 (40.8%) of them attended primary school (Table 1).

The results in Table 1 also indicate the prevalence of IPs with respective to sociodemographic characteristics of the study participants. The infection rate is high (54.2%) in pregnant women with age groups between 35 and 39 years. 52.3% from rural dwellers and 61.2% from those participants with illiterate educational status were infected.

### 3.2. Overall and Species-Specific Prevalence of Intestinal Parasitic Infection

The overall prevalence of IPs among pregnant women was 144 (43.5%). Eight different types of intestinal parasites were identified in this study (two protozoans and six helminthes species). *E. histolytica/dispar* has the highest prevalence (44.4%) among the identified parasites infected followed by *A. lumbricoides* (15.7%) and *G. lamblia* (11.8%). Nine of the cases were due to simultaneous infection by two different intestinal parasites. The highest prevalence of double infection was found in *G. lamblia* and *E. histolytica/dispar* (44.5%) followed by *A. lumbricoides* and *Taenia* species (33.3%) (Table 2).

### 3.3. Factors Associated with Intestinal Parasitic Infections

The association of IPs and the sociodemographic and medical history factors, hygiene, and environmental factors of the subjects are shown in Table 3. In the univariate analyses, significant associations were seen between intestinal parasites and residence (COR = 1.96, 95% CI: 1.26-3.05, *p* = 0.003), occupation with the greatest risk in those who were students (COR = 2.69, 95% CI: 1.24-5.85, *p* = 0.013), illiterate educational status (COR = 3.07, 95% CI: 1.47-6.44, *p* = 0.003), being in the second trimester (COR = 3.72, 95% CI: 1.47-9.4), *p* = 0.006), and being in the third trimester (COR = 3.70, 95% CI: 1.39–9.87), *p* = 0.009). In addition, the use of spring water was riskier than the use of tap water (COR = 3.44, 95% CI: 2.01-5.88, *p* ≤ 0.001) (Table 3).

The results of the multivariable logistic analysis showed that being a student (AOR = 3.35, 95% CI: 1.01-11.09, *p* = 0.047), second trimester (AOR = 3.94, 95% CI: 1.46-10.64, *p* = 0.007), third trimester (AOR = 3.32, 95% CI: 1.15-9.6, *p* = 0.027), and using spring water for drinking (AOR = 2.91, 95% CI: 1.62-5.22, *p* ≤ 0.001) were significantly associated with IPIs (Table 3).

## 4. Discussion

The prevalence of IPIs in the present study was 43.5%. It was consistent with previous studies conducted in Ethiopia such as 43.5% in Butajira [25], 43.8% in Lalo Kile District [21], and 41% in Gilgel Gibe dam area, South West Ethiopia [26], and other countries such as 43.4% in Ibadan, Nigeria [27], and 43.8% in Gabon [28]. However, our finding was lower than the findings of other similar types of studies reported from Ethiopia (70.6%) [17], Kenya (76.2%) [29], Thailand (70%) [30], and Venezuela (73.9%) [6] but was higher when compared to findings reported from Hossana, Southern Ethiopia (29.5%) [22], Bahir Dar, Northwest Ethiopia (31.5%) [15], Anbesame health center, Northwest Ethiopia (21.1%) [31], West Gojjam Zone, Northwest Ethiopia (37.3%) [19], and Kitale District, Kenya (13.8%) [8]. The existence of such variations may be due to the differences in practices of personal hygiene, environmental sanitation, health promotion practices, sociodemographic features, and geographical variation of the participants; the methods employed for stool examination and the time of study may have also contributed to the variation [15, 18, 19, 32].

The most identified parasite in this study was *Entamoeba histolytica/dispar* (44.4%) followed by *Ascaris lumbricoides* (15.7%) which was quite different from the similar study conducted in Bahir Dar, Northwest Ethiopia, where *Giardia lamblia* (13.3%) was the highest followed by *E. histolytica/dispar* (7.8%) [15]. On the other hand, different reports from Ethiopia [16], Venezuela [6], and Gabon [28] revealed that the commonest parasite was *A. lumbricoides*. Such variation could be related to the local environmental factors of the different areas or the behavioral practices of the people concerned and the type of the parasite studied (helminthes only in some studies) [17, 25, 26].

The *E. histolytica/dispar* prevalence (44.4%) from this study was comparable with the prevalence reported in West Gojjam Zone, Northwest Ethiopia (40.8%) [19]. However, it was higher than the report from Gondar Town (2.9%) [13], Venezuela (12%) [6], and Northwest Ethiopia (7.8%) [15]. The prevalence of *A. lumbricoides* (15.7%) was comparable with a previous study in Gilgel Gibe dam area (15%) [26] but lower than that of other works such as 57% in [6] and 32.7% in [17] and higher than that of other works such as 6.5% in [8], 2.9% in [13], 7.3% in [21], and 8.8% in [25].

The 11.8% prevalence of *G. lamblia* identified in the present study was lower than that of another study (19.1%) [19] but higher than the findings from Lalo Kile District, Western Ethiopia (0.9%) [21], and Gondar Town, Northwest Ethiopia (1.8%) [13]. Furthermore, the 9.8% prevalence of Taenia species in the current study was higher than 0.8% in the finding from Northwest Ethiopia [15]. The prevalence of *E. vermicularis* (6.5%) was similar to 6.3% in Venezuela [6] but higher compared to 0.7% in Kenya [8] and 3.5% in Nigeria [27].

The prevalence of hookworm infection (5.9%) was lower compared to several studies conducted in Gilgel Gibe dam area, Southwest Ethiopia (29.4%) [26], and in Northwest Ethiopia (14.2%) [17], but it was consistent with the study done in Felege Hiwot Referral Hospital, Northwest Ethiopia (5.5%) [15]. These differences may be due to the difference in lifestyle, geographical area, cultural practices, implementation of various intervention strategies, and awareness in the prevention of parasitic disease [21, 32]. In the present study, the prevalence of double infections (2.7%) was almost comparable with the report (3.38%) [15] but lower than the report from Ghana (6.6%) [33]. Moreover, the double infection rate (2.7%) was much lower than the study done in Venezuela (46.9%) [6]. It has been shown that coinfections of intestinal parasites are very common in most of the surveys in Africa such that cases of multiple infections with nematodes (Ascaris, hookworms, and Trichuris) have been reported [26, 27]. This difference may be due to the differences in the study subjects, sociodemographic conditions of the society, and differences in the parasitological examination techniques [19, 21, 26].

In the current finding, parasitic infection was detected in 63 (35.8%) and 81 (52.3%) urban and rural pregnant women, respectively. This was relatively consistent with the previously reported study which indicated that rural dwellers were more exposed to intestinal parasitic infections [8, 19, 26]. The high prevalence of IPs in rural areas may be attributed to poor personal hygiene and low economic status. Further, pregnant women in many rural areas depend on pit latrines for waste disposal with no facilities for hand washing after defecation [8, 19].

Intestinal parasitic infection among pregnant women had a significant association with occupational status, trimester, and source of drinking water. The odds of IPIs were 3.35-fold higher among pregnant women who were students compared to those who were government employees. This could be due to the lack of hand washing facilities in schools and the health-seeking behavior of employed pregnant women [17]. On the other hand, women in their second and third trimester of pregnancy had increased odds of infection 3.94 and 3.32 times, respectively. This result was in accordance with the previous study conducted in Ethiopia [15] where the odds of intestinal parasitic infection were increased by 22% in pregnant women in their second trimester. This finding was also in line with previous studies conducted in Kenya [33] and Benin [34] which showed an increased prevalence of intestinal parasite infections in the second and third trimester of pregnancy. In our study, using spring water as a source of drinking water was significantly associated with IPIs. This finding is in line with a previous study in Northwest Ethiopia [31]. This might be due to the seasonal flooding and possible latrine overflow of human waste into drinking water sources [8].

## 5. Limitation of the Study

Due to budget shortage, this study was done by the wet mount and formol ether concentration technique only which may underestimate the prevalence of IPIs. Additionally, we were unable to differentiate particular species of hookworms, *Taenia*, and *E. histolytica/dispar*.

This study is also limited by the fact that the prevalence of intestinal parasitic infection was based on a single fecal sample instead of the ideal three consecutive samples which may result in underestimation of the infections.

## 6. Conclusion and Recommendations

A high prevalence of intestinal parasites was observed in pregnant women. *E. histolytica/dispar* and *A. lumbricoides* were the most dominant parasites from protozoans and helminthes, respectively. Being a student, second and third trimester, and using spring water for drinking were associated factors with IPIs. Therefore, improving the sanitation of the students and providing clean drinking water for the women should be strengthened. Screening women and providing health education during their antenatal care (ANC) visits are recommended.

## Figures and Tables

**Table 1:** Sociodemographic characteristics of pregnant women with respective to IPs in Woreilu Health Center, Woreilu, Northeast Ethiopia, from October 2018 to February 2019.

Variables	Classification	Total *N* (%)	Negative *N* (%)	Positive *N* (%)
Age (years)	15-19	35 (10.6)	20 (57.1)	15 (42.9)
	20-24	112 (33.8)	70 (62.5)	42 (37.5)
	25-29	91 (27.5)	49 (53.8)	42 (46.2)
	30-34	55 (16.6)	29 (52.7)	26 (47.3)
	35-39	24 (7.3)	11 (45.8)	13 (54.2)
	≥40	14 (4.2)	8 (57.1)	6 (42.9)

Residence	Urban	176 (53.2)	113 (64.2)	63 (35.8)
	Rural	155 (46.8)	74 (47.7)	81 (52.3)

Marital status	Single	39 (11.8)	19 (48.7)	20 (51.3)
	Married	273 (82.5)	162 (59.3)	111 (40.7)
	Divorced	14 (4.2)	3 (21.4)	11 (78.6)
	Widowed	5 (1.5)	3 (60)	2 (40)

Occupation	Government employee	70 (21.1)	47 (67.1)	23 (32.9)
	Private	64 (19.3)	37 (57.8)	27 (42.2)
	Housewife	153 (46.3)	84 (54.9)	69 (45.1)
	Student	44 (13.3)	19 (43.2)	25 (56.8)

Education	Illiterate	67 (20.2)	26 (38.8)	41 (61.2)
	Primary	135 (40.8)	77 (57)	58 (43)
	Secondary	73 (22.1)	47 (64.4)	26 (35.6)
	Preparatory and above	56 (16.9)	37 (66.1)	19 (33.9)

**Table 2:** Frequency of occurrence of intestinal parasites in 144 positive stool samples and coinfections among pregnant women in Woreilu Health Center, Woreilu, Northeast Ethiopia, from October 2018 to February 2019.

	*N* (%)
Intestinal parasites	
*E. histolytica/dispar*	68 (44.4)
*A. lumbricoides*	24 (15.7)
*G. lamblia*	18 (11.8)
*Taenia* species	15 (9.8)
*E. vermicularis*	10 (6.5)
Hookworm	9 (5.9)
*S. mansoni*	6 (3.9)
*H. nana*	3 (2)
Total	153 (100)
Coinfections	
*G. lamblia+E. histolytica/dispar*	4 (44.5)
*A. lumbricoides*+Taenia species	3 (33.3)
*A. lumbricoides*+hookworm	1 (11.1)
*G. lamblia+H. nana*	1 (11.1)
Total	9 (100)

**Table 3:** Logistic regression analysis of intestinal parasitic infection across sociodemographic and other associated risk factors among pregnant women in Woreilu Health Center, Woreilu, Northeast Ethiopia from October 2018 to February 2019.

Variables	Classification	COR (95% CI)	*p* value	AOR (95% CI)	*p* value
Age (years)	15-19	1 (0.29-3.49)	1.000	0.44 (0.08-2.48)	0.356
	20-24	0.80 (0.26-2.47)	0.698	0.63 (0.14-2.84)	0.549
	25-29	1.14 (0.37-3.56)	0.818	0.96 (0.24-3.89)	0.953
	30-34	1.2 (0.37-3.9)	0.768	0.87 (0.23-3.31)	0.835
	35-39	1.58 (0.42-5.95)	0.502	2.15 (0.51-9.16)	0.299
	≥40	1		1	

Residence	Urban	1		1	
	Rural	1.96 (1.26, 3.05)	0.003	1.24 (0.69-2.24)	0.471

Marital status	Single	1		1	
	Married	0.65 (0.33-1.28)	0.211	0.86 (0.37-2.02)	0.735
	Divorced	3.48 (0.84-14.45)	0.086	3.87 (0.79-18.72)	0.093
	Widowed	0.63 (0.09-4.22)	0.637	0.70 (0.08-5.83)	0.742

Occupation	Government	1		1	
	Private	1.49 (0.74-3.01)	0.266	1.07 (0.41-2.78)	0.885
	Housewife	1.68 (0.93-3.03)	0.086	1.13 (0.44-2.91)	0.792
	Student	2.69 (1.24-5.85)	0.013	3.35 (1.01-11.09)	0.047^∗^

Education	Illiterate	3.07 (1.47-6.44)	0.003	2.61 (0.85-8.01)	0.09
	Primary	1.47 (0.77-2.81)	0.248	1.11 (0.42-2.95)	0.840
	Secondary	1.08 (0.52-2.24)	0.842	0.81 (0.32-2.06)	0.652
	Preparatory& above	1		1	

Gravidity	Primigravidae	0.79 (0.47-1.36)	0.403	1.52 (0.54-4.27)	0.432
	Bigravidae	1.06 (0.59-1.89)	0.832	1.89 (0.82-4.38)	0.134
	Multigravidae	1		1	

Trimester	1^st^	1		1	
	2^nd^	3.72 (1.47-9.4)	0.006	3.94 (1.46-10.64)	0.007^∗^
	3^rd^	3.70 (1.39–9.87)	0.009	3.32 (1.15-9.6)	0.027^∗^

Source of drinking water	Tap water	1		1	
	Well	2.02 (0.79-5.17)	0.142	1.63 (0.61-4.39)	0.334
	Spring	3.44 (2.01-5.88)	≤0.001	2.91 (1.62-5.22)	≤0.001^∗^

Availability of toilet	No	3.06 (1.48-6.34)	0.003	2.14 (0.91-5.05)	0.083
	Yes	1		1	

Hand wash after toilet	No	1.67 (0.64-4.35)	0.293	0.73 (0.23-2.31)	0.596
	Yes	1		1	

Shoe wearing	No	1.30 (0.18-9.36)	0.793	0.860 (0.09-7.78)	0.894
	Yes	1		1	

Eating raw meat	No	1		1	
	Yes	0.92 (0.57-1.47)	0.718	1.28 (0.71-2.28)	0.414

Eating raw vegetables or fruit	No	1		1	
	Yes	0.83 (0.489-1.41)	0.490	0.781 (0.42-1.45)	0.433

Hand wash with soap before food meal	No	0.77 (0.05-12.4)	0.853	0.52 (0.03-9.26)	0.653
	Yes	1		1	

Soil eating habit	No	1		1	
	Yes	1.5 (0.93-2.41)	0.094	1.27 (0.75-2.14)	0.379

## Data Availability

The data that support the findings of this study are available upon reasonable request from the corresponding author.

## Abbreviations

ANC: Antenatal care center
IPIs: Intestinal parasitic infections
WHO: World Health Organization.

## Supplementary Materials

Supplementary Materials S1_File: ethical clearance letter.
