# Supplementary material for: Prevalence of Intestinal Parasitic Infections and Their Associated Risk Factors among Pregnant Women Attending Antenatal Care Center at Woreilu Health Center, Woreilu, Northeast Ethiopia
Source: J Parasitol Res. 2022 Apr 11;2022:5242252. doi: 10.1155/2022/5242252 (PMC9017482; doi:10.1155/2022/5242252)
Supplement: Supplementary Materials — S1_File: ethical clearance letter. [file 5242252.f1.pdf]

ወሎ ዩኒቨርሲቲ
በህክምናና ጤና ሳይንስ ኮሌጅ
የምርምር፣ማህበረሰብ አገልግሎትና
ድህረ-ምረቃ ማስተባበሪያ ጽ/ቤት
ደሴ፣ኢትዮጵያ

Wollo University
College of Medicine and Helath Science
Research, Community Service and
Post graduate Coordinating Office
Dessie, Ethiopia

ቁጥር CMHS-RCSPC/102/18
Ref. No.
ቀን September 20, 2018
Date

To: Mr. Daniel Gebretsadik

Subject: Ethical Review

The Ethical Review committee of College of Medicine and Health Science, Wollo university has thoroughly examined your research proposal entitled '**Prevalence of intestinal parasitic infections and associated risk factors among pregnant women attending at Antenatal care Center of Woreilu health center, Woreilu, Northeast Ethiopia.'** The committee **approved** your research proposal **with Recommendation**. In the meantime, committee has also learnt that the comments and suggestions are rectified as per the recommendations. Hence, the committee has officially allowed you to proceed your research activity. The previous decision of the committee, comments and suggestions are attached here with this letter and thereby please find all.

Regards,

መንግስቱ አባተ በላይ
Mengistu Abate Belay
የምርምር፣ማህበረሰብ አገልግሎት
እና የድህረ-ምረቃ አስተባባሪ
Research, Community Service &
Post-graduate Studies Coordinator

የፖ.ሣ.ቁ.
P.O.Box- 1145

ቴሌ ግራም ጤ.ኮ.
Fax- 251 3324352

ስልክ
Telephone- 251 338110288

መልስ ሲጽፉልን የእኛን ቁጥር ይጥቀሱ።
In replying, please quote our Ref. No.
